# Complete Genome Sequence of Enterobacter Phage vB_EcRAM-01, a New *Pseudotevenvirus* against the Enterobacter cloacae Complex

**DOI:** 10.1128/mra.00045-22

**Published:** 2022-05-10

**Authors:** Ednner E. Victoria-Blanco, Jean Pierre González-Gómez, Evelia Quiroz, Alexander A. Martínez, Claudia González, Nohelia Castro del Campo, Cristóbal Chaidez-Quiroz, Jordi Querol-Audi, Alex Omar Martínez-Torres

**Affiliations:** a Human Microbiology Department, School of Medicine, University of Panama, Panama City, Panama; b Biomedical Sciences Graduate Program, School of Medicine, University of Panama, Panama City, Panama; c Applied and Experimental Microbiology Laboratory (LAMEXA), University of Panama, Panama City, Panama; d Water Microbiology Laboratory (LAMA), University of Panama, Panama City, Panama; e Laboratorio Nacional para la Investigación en Inocuidad Alimentaria (LANIIA), Centro de Investigación en Alimentación y Desarrollo, A.C. (CIAD), Sinaloa, México; f Genomics and Proteomics Research Department, Gorgas Memorial Institute of Health Studies, Panama, Panama; g Sistema Nacional de Investigación (SNI), Secretaría Nacional de Ciencia, Tecnología e Innovación SENACYT, Panama, Panama; Loyola University Chicago

## Abstract

Here, we present the complete genome sequence of Enterobacter phage vB_EcRAM-01, isolated from waters of the Río Abajo river, in Panama City, Panama. This phage has deployed lytic activity against the Enterobacter cloacae complex, a pathogen of clinical importance in intensive care units. It belongs to the *Myoviridae* family and has a double-stranded DNA genome that is 178,477 bp long and contains 293 open reading frames (ORFs).

## ANNOUNCEMENT

The Enterobacter cloacae complex has been classified by the World Health Organization (WHO) as a priority for antimicrobial research and development (1). In intensive care units, phage therapy has become a viable option for treating multidrug-resistant bacteria, among which E. cloacae represents a serious threat (2, 3).

The Enterobacter phage vB_EcRAM-01 was isolated from waters of the Rio Abajo river in Panama City, Panama (coordinates, 9°00′55.2″N 79°29′25.6″W), using E. cloacae strain ATCC 23355 as a propagation host, which was grown previously in tryptic soy broth at 37°C for 24 h (4, 5). Phage isolation, propagation, and purification were performed using the soft-agar overlay method (6). DNA extraction was performed using the QIAamp MinElute virus spin kit (Qiagen, Valencia, CA, USA), according to the manufacturer’s instructions. The DNA libraries were prepared using the Nextera XT DNA library preparation kit (Illumina, San Diego, CA, USA) through tagmentation, PCR amplification, PCR cleanup, and library normalization. Genome sequencing was performed with the Illumina MiSeq platform (2 × 250-bp paired-end protocol, 300 cycles). Raw reads (629,372 reads in total) were checked for quality using FastQC (www.bioinformatics.babraham.ac.uk/projects/fastqc) and trimmed using Trimmomatic v0.39 (7). The genome was assembled through SPAdes v3.15.1 (8), resulting in a single contig with 8.96-fold coverage. We mapped the reads against the resulting contig to confirm sequence ends using Bowtie (9). Alignments with closely related phages showed the presence of the same annotated features which indicates this single contig represents the complete genome. The genome was initially annotated with the fast annotation algorithm using RASTk v2.0 subsystem technology (10–12). The putative open reading frames (ORFs) were verified using GeneMarkS v4.28 (13, 14) and Glimmer v3.02 (15, 16).

The ORF functions were annotated using the protein basic local alignment search tool (blastp) of the NCBI server (https://blast.ncbi.nlm.nih.gov/Blast.cgi) based on a search of the nonredundant protein sequence database (17), HHpred using the structural/domain database (https://toolkit.tuebingen.mpg.de/tools/hhpred) (18, 19), and HMMER v2.41.1 through the HMM database (20, 21).

vB_EcRAM-01 has a 178,477-bp genome containing 293 genes, of which 86 have a predicted function associated with morphology, inactivation, cell adsorption, DNA injection, and host lysis. No resistance (ResFinder 4.1, https://cge.cbs.dtu.dk/services/ResFinder/) (22–24), virulence (VirulenceFinder 2.0, https://cge.cbs.dtu.dk/services/VirulenceFinder/) (25, 26), or allergenicity-related (AllergenOnline, http://www.allergenonline.org/databasefasta.shtml) (27) genes were identified. The raw reads were assembled, automatically annotated, and manually curated. This genome codes for 2 tRNAs, namely, a tRNA-Met (cat) and a tRNA-Gly (tcc). Enterobacter phage vB_EcRAM-01 is most closely related to *Cronobacter* phage vB_CsaM_leE (GenBank accession number NC_048646.1) with 90.11% nucleotide sequence identity determined by pyani v0.2.11 (28). Therefore, Enterobacter phage vB_EcRAM-01 is a new member of the *Pseudotevenvirus* genus within the *Tevenvirinae* subfamily and *Myoviridae* family, according to the genus demarcation criteria (>70% nucleotide identity over the full genome length) recommended by the ICTV Bacterial and Archaeal Viruses Subcommittee (29).

vB_EcRAM-01 exhibited specific litic activity against 19 clinical E. cloacae isolates.

The complete annotation of the vB_EcRAM-01 genome can be found in the GenBank database (30).

### Data availability.

The complete sequence of Enterobacter phage vB_EcRAM-01 has been deposited in GenBank under accession number OL551674 and BioSample accession SAMN23426491. Raw data can be accessed through SRA number SRR18344380.

## References

[1] WHO. 2017. Global priority list of antibiotic-resistant bacteria to guide research, discovery, and development of new antibiotics. WHO, Geneva, Switzerland.

[2] Abedon ST. 2017. Information phage therapy research should report. Pharmaceuticals 10:43. doi:10.3390/ph10020043. PMC5490400 28468287

[3] Abedon ST (ed). 2008. Bacteriophage ecology: population growth, evolution, and impact of bacterial viruses, 1 ed. Cambridge University Press, Cambridge, England.

[4] Gaviria AG, González de SM, Castaño OJ. 2012. Técnica para aislamiento de bacteriófagos específicos para *E. coli* DH5α a partir de aguas residuales. Rev MVZ Córdoba 17:2852–2860. doi:10.21897/rmvz.253.

[5] Meza A. 2016. Aislamiento y caracterización de bacteriófagos para cepas nosocomiales DE Acinetobacter baumannii de Lima e Iquitos, Perú 2014–2015. Universidad Católica De Santa María, Lima, Peru.

[6] Piuri M. 2019. Los bacteriófagos: del genoma al metagenoma. Departamento de Quimica Biológica Universidad de Buenos Aires, Argentina.

[7] Bolger AM, Lohse M, Usadel B. 2014. Trimmomatic: a flexible trimmer for Illumina sequence data. Bioinformatics 30:2114–2120. doi:10.1093/bioinformatics/btu170. PMC4103590 24695404

[8] Bankevich A, Nurk S, Antipov D, Gurevich AA, Dvorkin M, Kulikov AS, Lesin VM, Nikolenko SI, Pham S, Prjibelski AD, Pyshkin AV, Sirotkin AV, Vyahhi N, Tesler G, Alekseyev MA, Pevzner PA. 2012. SPAdes: a new genome assembly algorithm and its applications to single-cell sequencing. J Comput Biol 19:455–477. doi:10.1089/cmb.2012.0021. 22506599 PMC3342519

[9] Langmead B, Trapnell C, Pop M, Salzberg SL. 2009. Ultrafast and memory-efficient alignment of short DNA sequences to the human genome. Genome Biol 10:R25. doi:10.1186/gb-2009-10-3-r25. 19261174 PMC2690996

[10] Brettin T, Davis JJ, Disz T, Edwards RA, Gerdes S, Olsen GJ, Olson R, Overbeek R, Parrello B, Pusch GD, Shukla M, Thomason JA, Stevens R, Vonstein V, Wattam AR, Xia F. 2015. RASTtk: a modular and extensible implementation of the RAST algorithm for building custom annotation pipelines and annotating batches of genomes. Sci Rep 5:8365. doi:10.1038/srep08365. 25666585 PMC4322359

[11] Overbeek R, Olson R, Pusch GD, Olsen GJ, Davis JJ, Disz T, Edwards RA, Gerdes S, Parrello B, Shukla M, Vonstein V, Wattam AR, Xia F, Stevens R. 2014. The SEED and the Rapid Annotation of microbial genomes using Subsystems Technology (RAST). Nucleic Acids Res 42:D206–D214. doi:10.1093/nar/gkt1226. 24293654 PMC3965101

[12] Aziz RK, Bartels D, Best AA, DeJongh M, Disz T, Edwards RA, Formsma K, Gerdes S, Glass EM, Kubal M, Meyer F, Olsen GJ, Olson R, Osterman AL, Overbeek RA, McNeil LK, Paarmann D, Paczian T, Parrello B, Pusch GD, Reich C, Stevens R, Vassieva O, Vonstein V, Wilke A, Zagnitko O. 2008. The RAST server: Rapid Annotations using Subsystems Technology. BMC Genomics 9:75. doi:10.1186/1471-2164-9-75. 18261238 PMC2265698

[13] Delcher AL, Bratke KA, Powers EC, Salzberg SL. 2007. Identifying bacterial genes and endosymbiont DNA with Glimmer. Bioinformatics 23:673–679. doi:10.1093/bioinformatics/btm009. 17237039 PMC2387122

[14] Salzberg SL, Delcher AL, Kasif S, White O. 1998. Microbial gene identification using interpolated Markov models. Nucleic Acids Res 26:544–548. doi:10.1093/nar/26.2.544. 9421513 PMC147303

[15] Besemer J, Borodovsky M. 2005. GeneMark: Web software for gene finding in prokaryotes, eukaryotes and viruses. Nucleic Acids Res 33:W451–W454. doi:10.1093/nar/gki487. 15980510 PMC1160247

[16] Besemer J, Lomsadze A, Borodovsky M. 2001. GeneMarkS: a self-training method for prediction of gene starts in microbial genomes. Implications for finding sequence motifs in regulatory regions. Nucleic Acids Res 29:2607–2618. doi:10.1093/nar/29.12.2607. 11410670 PMC55746

[17] Altschul SF, Madden TL, Schäffer AA, Zhang J, Zhang Z, Miller W, Lipman DJ. 1997. Gapped BLAST and PSI-BLAST: a new generation of protein database search programs. Nucleic Acids Res 25:3389–3402. doi:10.1093/nar/25.17.3389. 9254694 PMC146917

[18] Zimmermann L, Stephens A, Nam S-Z, Rau D, Kübler J, Lozajic M, Gabler F, Söding J, Lupas AN, Alva V. 2018. A completely reimplemented MPI bioinformatics toolkit with a new HHpred server at its core. J Mol Biol 430:2237–2243. doi:10.1016/j.jmb.2017.12.007. 29258817

[19] Hildebrand A, Remmert M, Biegert A, Söding J. 2009. Fast and accurate automatic structure prediction with HHpred. Proteins 77:128–132. doi:10.1002/prot.22499. 19626712

[20] Potter SC, Luciani A, Eddy SR, Park Y, Lopez R, Finn RD. 2018. HMMER Web server: 2018 update. Nucleic Acids Res 46:W200–W204. doi:10.1093/nar/gky448. 29905871 PMC6030962

[21] Finn RD, Clements J, Eddy SR. 2011. HMMER Web server: interactive sequence similarity searching. Nucleic Acids Res 39:W29–W37. doi:10.1093/nar/gkr367. 21593126 PMC3125773

[22] Bortolaia V, Kaas RS, Ruppe E, Roberts MC, Schwarz S, Cattoir V, Philippon A, Allesoe RL, Rebelo AR, Florensa AF, Fagelhauer L, Chakraborty T, Neumann B, Werner G, Bender JK, Stingl K, Nguyen M, Coppens J, Xavier BB, Malhotra-Kumar S, Westh H, Pinholt M, Anjum MF, Duggett NA, Kempf I, Nykäsenoja S, Olkkola S, Wieczorek K, Amaro A, Clemente L, Mossong J, Losch S, Ragimbeau C, Lund O, Aarestrup FM. 2020. ResFinder 4.0 for predictions of phenotypes from genotypes. J Antimicrob Chemother 75:3491–3500. doi:10.1093/jac/dkaa345. 32780112 PMC7662176

[23] Zankari E, Allesøe R, Joensen KG, Cavaco LM, Lund O, Aarestrup FM. 2017. PointFinder: a novel web tool for WGS-based detection of antimicrobial resistance associated with chromosomal point mutations in bacterial pathogens. J Antimicrob Chemother 72:2764–2768. doi:10.1093/jac/dkx217. 29091202 PMC5890747

[24] Camacho C, Coulouris G, Avagyan V, Ma N, Papadopoulos J, Bealer K, Madden TL. 2009. BLAST+: architecture and applications. BMC Bioinformatics 10:421. doi:10.1186/1471-2105-10-421. 20003500 PMC2803857

[25] Joensen KG, Scheutz F, Lund O, Hasman H, Kaas RS, Nielsen EM, Aarestrup FM. 2014. Real-time whole-genome sequencing for routine typing, surveillance, and outbreak detection of verotoxigenic Escherichia coli. J Clin Microbiol 52:1501–1510. doi:10.1128/JCM.03617-13. 24574290 PMC3993690

[26] Malberg Tetzschner AM, Johnson JR, Johnston BD, Lund O, Scheutz F. 2020. In silico genotyping of Escherichia coli isolates for extraintestinal virulence genes by use of whole-genome sequencing data. J Clin Microbiol 58:e01269-20. doi:10.1128/JCM.01269-20. 32669379 PMC7512150

[27] Goodman RE, Ebisawa M, Ferreira F, Sampson HA, van Ree R, Vieths S, Baumert JL, Bohle B, Lalithambika S, Wise J, Taylor SL. 2016. AllergenOnline: a peer-reviewed, curated allergen database to assess novel food proteins for potential cross-reactivity. Mol Nutr Food Res 60:1183–1198. doi:10.1002/mnfr.201500769. 26887584

[28] Pritchard L, H Glover R, Humphris S, G Elphinstone J, K Toth I. 2016. Genomics and taxonomy in diagnostics for food security: soft-rotting enterobacterial plant pathogens. Anal Methods 8:12–24. doi:10.1039/C5AY02550H.

[29] Turner D, Kropinski AM, Adriaenssens EM. 2021. A roadmap for genome-based phage taxonomy. Viruses 13:506. doi:10.3390/v13030506. 33803862 PMC8003253

[30] Karsch-Mizrachi I, Nakamura Y, Cochrane G, International Nucleotide Sequence Database Collaboration. 2012. The International Nucleotide Sequence Database Collaboration. Nucleic Acids Res 40:D33–D37. doi:10.1093/nar/gkr1006. 22080546 PMC3244996

